# Soil-transmitted helminth reinfection four and six months after mass drug administration: results from the delta region of Myanmar

**DOI:** 10.1371/journal.pntd.0006591

**Published:** 2019-02-15

**Authors:** Julia C. Dunn, Alison A. Bettis, Nay Yee Wyine, Aye Moe Moe Lwin, Aung Tun, Nay Soe Maung, Roy M. Anderson

**Affiliations:** 1 Department of Infectious Disease Epidemiology, School of Public Health, Faculty of Medicine, Imperial College London, London, United Kingdom; 2 London Centre for Neglected Tropical Disease Research, London, United Kingdom; 3 University of Public Health, Yangon, Myanmar; 4 Ministry of Health and Sports, Nyapyitaw, Myanmar; University of New South Wales Faculty of Medicine, AUSTRALIA

## Abstract

**Background:**

Mass drug administration (MDA), targeted at school-aged children (SAC) is the method recommended by the World Health Organization for the control of morbidity induced by soil-transmitted helminth (STH) infection in endemic countries. However, MDA does not prevent reinfection between treatment rounds and research suggests that only treating SAC will not be sufficient to bring prevalence to low levels and possibly interrupt transmission of STH. In countries with endemic infection, such as Myanmar, the coverage, who is targeted, and rates of reinfection will determine how effective MDA is in suppressing transmission in the long-term.

**Methods/principal findings:**

In this paper, data from an epidemiological study on STH, comprising three surveys conducted between June 2015 and June 2016 in the delta region of Myanmar, are analysed to determine how STH prevalence and intensity in the study community changes over the course of a year, including reinfection after two MDA rounds in which the whole study sample (all age groups, n = 523) were treated with albendazole. Prevalence in the first survey (August 2015) was 27.92% for any STH, 5.54% for *Ascaris lumbricoides*, 17.02% for *Trichuris trichiura* and 9.75% for hookworm. Over the year (survey one to survey three), prevalence of any STH decreased by 8.99% (*P* < 0.001) and mean EPG significantly decreased for *T*. *trichiura* (*P* < 0.01) and hookworm (*P* < 0.001). Risk ratios (RRs) for a four-month reinfection period (August to December) were statistically significant and were below one, indicating that STH prevalence had not bounced back to the prevalence levels recorded immediately prior to the last round of treatment (any STH RR = 0.67, 95% CI 0.56–0.81; *A*. *lumbricoides* RR = 0.31, 95% CI 0.16–0.59; *T*. *trichiura* RR = 0.70, 95% CI 0.55–0.88; hookworm RR = 0.69, 95% CI 0.50–0.95). The only statistically significant RR for the six-month reinfection period (December to June) was for *A*. *lumbricoides* infection in SAC (RR = 2.67, 95% CI 1.37–5.21). All six-month RRs were significantly higher than four-month RRs (*P* < 0.05). Evidence of predisposition to infection (low and high), as measured by the Kendall Tau-b statistic, was found for all species overall and within most age groups stratifications, except for hookworm infection in preschool-aged children.

**Conclusions/significance:**

This study demonstrates that, for certain demographic groups, a six-month gap between MDA in these communities is enough time for STH infection to return to STH prevalence levels recorded immediately before the previous MDA round, and that on average the same individuals are being consistently infected between MDA rounds.

## Author summary

Mass drug administration (MDA), treating either whole communities or targeted groups without a prior diagnosis, is used as a control strategy for many neglected tropical diseases, including soil-transmitted helminth (STH) infection. MDA takes place at set intervals, aiming to reduce morbidity caused by the target disease. Research and policy focus is also increasingly considering the potential of interrupting STH transmission, leading to elimination. In this study we measure STH infection in two villages in the delta region of Myanmar over the course of a year, both before and after MDA rounds, to quantify the effect of treatment on infection and to identify groups with persistent infections. We found that whilst overall prevalence of STH infection decreased over the year, intensity of infection, measured by eggs per gram of faeces, did not significantly decrease. We also found evidence to suggest that particular people are predisposed to STH infection. This is possibly due to non-compliance to MDA, or behavioural and social factors. The findings presented here will provide evidence to support continuing Myanmar’s MDA programme for STH control and using accurate diagnostics to identify and target “predisposed” people for sustained treatment.

## Introduction

Soil-transmitted helminth infections (STHs) are classified by the World Health Organization (WHO) as neglected tropical diseases (NTDs). Approximately 1.4 billion people worldwide are estimated to be infected with at least one of the main STHs (*Ascaris lumbricoides*, *Trichuris trichiura*, *Ancylostoma duodenale*, *Necator americanus*) [1]. Endemic countries carry out mass drug administration (MDA) campaigns to control STH infections with the goals of reducing STH prevalence and intensity of infection to a level where there is a low risk of morbidity in children [2,3]. The WHO recommends that MDA is carried out annually or biannually, targeting school-aged children (SAC, 5–14 years old) as they are at the highest risk of morbidity [3,4]. A goal set by the WHO for STH control is to decrease the prevalence of medium and high intensity infections (MHII) in SAC to below 1% at which point STH morbidity has been eliminated as a public health problem in the area [2]. In 2017, this guideline was updated to include treatment of young children (12 to 23 months old), preschool-aged children (pre-SAC, 2–4 year olds), adolescent girls (10–19 year olds) and women of reproductive age (WRA, 15–45 year olds) [3]. Whilst MDA that targets pre-SAC and SAC can reduce morbidity in these groups, research indicates that MDA targeting all age groups (community-wide) is more effective at reducing STH prevalence and intensity in all groups, especially in hookworm-endemic areas [5,6], and to interrupt transmission [7–9].

Myanmar, in Southeast Asia, is endemic for STH. A WHO-led survey conducted in 2002–2003 found that 69.7% of the SAC sampled were infected with at least one STH (*A*. *lumbricoides* = 48.5%, *T*. *trichiura* = 57.5% and hookworm = 6.5%) and 18.2% of SAC had a medium/high intensity infection [10]. MDA programmes targeting STH and lymphatic filariasis (LF) have been conducted in Myanmar since 2003. The LF MDA programme is conducted under the Global Programme to Eliminate Lymphatic Filariasis (GPELF) and treats all age groups annually with albendazole and diethylcarbamazine (DEC) in December or January [11]. The STH MDA programme treats pre-SAC and 5–9 year olds (primary school children) annually, eight months after GPELF (August), with albendazole. A follow-up WHO survey, conducted in 2012, found that, after seven years of MDA, STH prevalence in SAC had decreased to 20.9% [12]. Coverage of the STH programme has been consistently high since 2006, national coverage of SAC was reported as 97.49% in 2016 [12,13]. There are few recent epidemiological studies on STH in Myanmar. In 2015, Htoon *et al*. reported 18.6% prevalence of *T*. *trichiura* in urban 6–8 year olds [14].

Anthelminthic drugs kill helminths within hosts, but do not prevent reinfection between MDA rounds [15,16]. Research into drug efficacy also suggests that a single dose of albendazole, as given in most countries’ MDA programmes, will not clear intestinal helminths, especially *T*. *trichiura* infections [17–20]. Therefore, as well as individuals gaining new infections after MDA, they may also be harbouring old infections not killed by previous treatment. Reinfection, or the change in STH prevalence and intensity over time, depends on multiple factors: the efficacy of the anthelminthics and coverage of MDA to effectively clear STH infections from all infected individuals in the population, the level of environmental contamination with eggs and larvae and an individual’s exposure to environmental contamination (behavioural and social factors) [21].

Those who are consistently reinfected with STH after clearing their infections with treatment are considered to be “predisposed” to infection [22]. Research is ongoing to determine what the underlying factors of predisposition are. They are likely to be a combination of genetic, immunological, environmental and behavioural factors [23–25]. Predisposition is usually defined as individuals who consistently reacquire infection to the same intensity class of infection (i.e. low, medium, high) as prior to treatment [26]. However, in low STH intensity populations, such as those that have undergone multiple years of MDA, the definition of predisposition could be widened to include all those who consistently harbour STH infections between MDA rounds. Being able to prioritise the groups of people that are consistently infected, despite MDA, would be highly beneficial to STH control programmes that are in the final years of MDA and are targeting the interruption of transmission [9,27]. A change in treatment policy may be desirable to target those predisposed to infection if they can easily be identified.

In this paper we describe and analyse the infection status of study participants in a longitudinal epidemiological study of STH in Myanmar that was conducted between June 2015 and June 2016. The aim of this analysis is to determine how the prevalence and intensity of STH infection changes over the course of a year under the influence of the STH and LF MDA programmes in Myanmar, both of which aim to significantly reduce STH prevalence and intensity (although this is a secondary aim for the LF MDA programme). We also investigate if there is evidence for predisposition to infection in the study sample when stratified by a variety of confounding variables including age and sex.

## Methods

### Ethics statement

**Fig 1:**
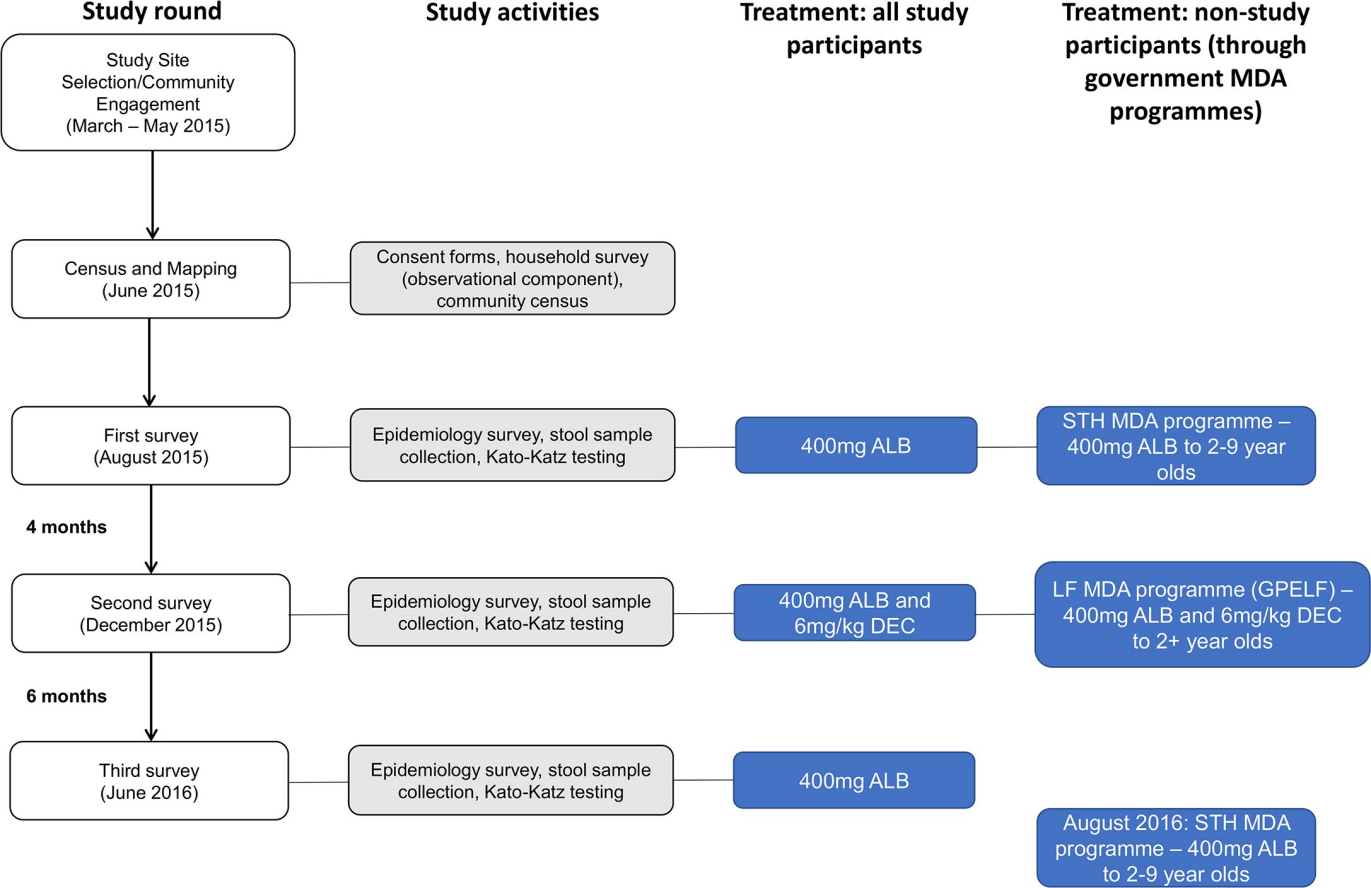
Flow diagram of data collection and study methods. ALB = albendazole, DEC = diethylcarbamazine citrate, GPELF = Global Programme to Eliminate Lymphatic Filariasis, LF = lymphatic filariasis, MDA = mass drug administration, STH = soil-transmitted helminths. Adapted with permission from the supplementary information of Dunn et al. (2017) [28].

Data were collected in an STH epidemiological study that has been detailed in a previous publication [28]. The study received ethical approval from the Imperial College Research Ethics Committee, Imperial College London, UK (ICREC– 15IC2667) and the Department of Medical Research Ethics Review Committee, Ministry of Health and Sports, Myanmar. The study was explained to each potential participant and consent was sought at this time. Adults (18 years and above) provided consent for themselves and for any children (under 18 years old) under their care (parents or guardians). Children aged 10–17 years old also signed assent forms. All participants in the study were offered anthelminthics regardless of their disease status and non-participants in the study were treated as usual by the governmental MDA programmes (Fig 1). Treatment was instructed to be directly observed, although this could not be guaranteed in all cases. All participants provided their own answers to questionnaires except children from 2–4 years old, for whom we received answers from a parent/guardian.

### Study sites and design

Data collection took place between June 2015 and June 2016 in Udo village, Taikkyi township, Yangon Region and Kyee Kan Theik village, Nyaung Don township, Ayeyarwaddy Region. Further details on the study sites, including environmental and socioeconomic information, are provided in a previous publication [28]. In June 2015, a demographic survey and census were completed in the two study villages. Participants that fulfilled the inclusion criteria (permanent residents of the village, provided informed written consent, above two years old, not pregnant/breastfeeding) were recruited for the study by random selection of households. For each randomly selected household, the number of eligible household residents were tallied, and selection of households continued until the required sample size had been reached. Participants completed questionnaires collecting data on participants’ socioeconomic status, household structure and access to water, sanitation and hygiene (WaSH) facilities. The study comprised three parasitology surveys in August 2015 (first survey–S1), December 2015 (second survey–S2) and June 2016 (third survey–S3) (Fig 1). Stool samples were collected from the participants in each parasitology survey and were assessed for STH infection by trained laboratory technicians using the Kato-Katz method [29]. Egg counts were multiplied by 24 to give eggs per gram of faeces (EPG) [30]. The participants and their stool samples were assigned unique identification (ID) codes to maintain confidentiality and to link results over all surveys. All study participants were treated with 400mg albendazole at each survey and were also treated with 6mg/kg DEC at S2. Since the study included participants from all age groups above two years old, this meant that participants over the age of nine years received two additional albendazole treatments (in S1 and S3) compared to the regular treatment schedule (Fig 1).

### Data

Data for the following analyses were from all participants who had a recorded Kato-Katz result from all three surveys. Overall, 523 participants (53.5% of total recruited participants, 19.0% of village populations) from 211 households (67.6% of total recruited households, 35.6% of total households in the villages) had Kato-Katz data from all three surveys (S1 Fig). Table 1 presents the age and sex breakdown of the study participants. Data from both villages were merged and analysed as one dataset.

**Table 1:** Demographic characteristics of study participants–individuals and households.

Characteristic		*n*	%
**Individuals**		523	100
Village	Udo village	211	40.34
	Kyee Kan Theik village	312	59.70
Sex	Male	233	44.55
	Female	290	55.45
Age group (years)	2–4	39	7.46
	5–14	114	21.80
	15–24	40	7.65
	25–39	124	23.71
	40+	206	39.34
Age group (WHO-defined)	Pre-SAC	39	7.46
	SAC	114	21.80
	Adults	370	70.75
**Households**		211	100
Village	Udo village	91	43.13
	Kyee Kan Theik village	120	56.87
Overall household size^*^	1–4	107	50.71
	5–8	96	45.5
	9+	8	3.79

*Including non-participants.

### Statistical analysis

RStudio (R version 3.0.1, Vienna, Austria) was used for the following statistical analyses and to create the figures. Participants were grouped into age groups as defined by the WHO: preschool-aged children (pre-SAC) are 2–4 year olds, school-aged children (SAC) are 5–14 year olds and adults are 15+ year olds [4]. Exact confidence intervals (95% two-sided) for mean prevalence were calculated using the Clopper-Pearson method [31]. Non-parametric mean EPG adjusted percentiles (95% two-sided, bias-corrected and accelerated—BCa) were calculated using bootstrapping methodology with the *“boot”* package. Risk ratios (RRs) were calculated by dividing the prevalence of infection in the later survey by the prevalence of infection in the earlier survey (e.g. RR = prevalence of *A*. *lumbricoides* in S2 / prevalence of *A*. *lumbricoides* in S1). RR confidence intervals were calculated by multiplying the standard error of the natural logarithm of the RR by the z-score, and adding or subtracting the value from the log RR [32]. The significance test for the differences between RRs was derived from a formula published by Altman and Bland, 2003 [33]. The WHO recommended intensity cut-offs were used to group individual EPG into low, medium and high intensity infections [2]. We used McNemar’s test to assess the differences in prevalence and Wilcoxon signed rank test to assess the differences in intensity over the surveys, the significance level was set at *P* ≤ 0.05. We used the Kendall rank correlation coefficient test to analyse the correlation between egg counts in different surveys, indirectly assessing predisposition to infection. The Kendall Tau-b value was chosen as it adjusts for tied ranks and the significance level set at *P* ≤ 0.05. Data from all participants were included in Kendall-Tau analysis, including those who were uninfected in multiple surveys. Kendall Tau-b values range between minus one (all pairs are discordant) and one (all pairs are concordant), a higher Tau-b value indicates more concordant than discordant pairs of individual egg counts and therefore higher overall correlation [34].

The study took place over the course of a year. Therefore, all participants will have aged one year during the study. Whilst there is a well-established relationship between age and STH infection, for simplicity we maintained the recorded ages for all participants at the age recorded in the first survey. This is assuming that age-related exposure did not drastically change over the course of a year. Also, we have maintained the usual WHO definition of SAC (5–14 years old), despite the fact that there is a different treatment frequency for 5–9 and 10–14 year olds in Myanmar. We have done this to align with how the WHO expects STH outcomes to be reported regarding infection prevalence and intensity by age grouping. We cannot guarantee that all infections were cleared after MDA. Therefore, “reinfection” in the case of our analysis does not necessarily refer to new infections picked up between MDA rounds, but rather changes in the number and proportion of infections between surveys.

## Results

### Reinfection–prevalence

Baseline (S1) prevalence was 27.92% for any STH, 5.54% for *A*. *lumbricoides*, 17.02% for *T*. *trichiura* and 9.75% for hookworm (Table 2). From S1 to S2 prevalence of any STH fell by 8.99% and the reduction was statistically significant (*P* < 0.001), there was no change in prevalence from S1 to S2 (not statistically significant) and therefore the change in prevalence over the study year (S1 to S3) was also significant (*P* < 0.001). The reductions in prevalence of each STH separately, were statistically significant from S1 to S2 and from S1 to S3 (*P* < 0.05). There were no statistically significant changes in prevalence from S2 to S3. In the final survey (S3), prevalence of infection with any STH was 28.07% in SAC.

**Table 2:** Number of positive individuals (n), prevalence (%) and infection intensity of each soil-transmitted helminth species (overall n = 523).

	Any STH		*Ascaris lumbricoides*			*Trichuris trichiura*			Hookworm		
	n	% (95% CI)*	n	% (95% CI)	Mean EPG (95% CI)	n	% (95% CI)	Mean EPG (95% CI)	n	% (95% CI)	Mean EPG (95% CI)
**Survey 1 (Aug 2015)**	146	27.92 (24.64–32.64)	29	5.54 (3.83–8.03)	649.42 (370.35–1119.4)	89	17.02 (14.2–20.95)	73.56 (46.99–124.35)	51	9.75 (7.51–12.89)	40.2 (22.39–93.3)
**Survey 2 (Dec 2015)**	99	18.93 (16.00–23.03)	9	1.72 (0.81–3.31)	478.12 (87.36–1967.6)	62	11.85 (9.41–15.25)	24.32 (13.77–54.47)	35	6.69 (4.81–9.38)	314.94 (10.69–1533.86)
**Survey 3 (Jun 2016)**	99	18.93 (16.00–23.03)	13	2.49 (1.36–4.3)	670.35 (315.2–1392.34)	62	11.85 (9.41–15.25)	41.07 (24.36–72.28)	29	5.54 (3.83–8.03)	11.47 (6.38–24.78)

*% represents the percentage positive in each group. CI = Confidence interval. EPG = eggs per gram of faeces. STH = soil-transmitted helminth

**Fig 2:**
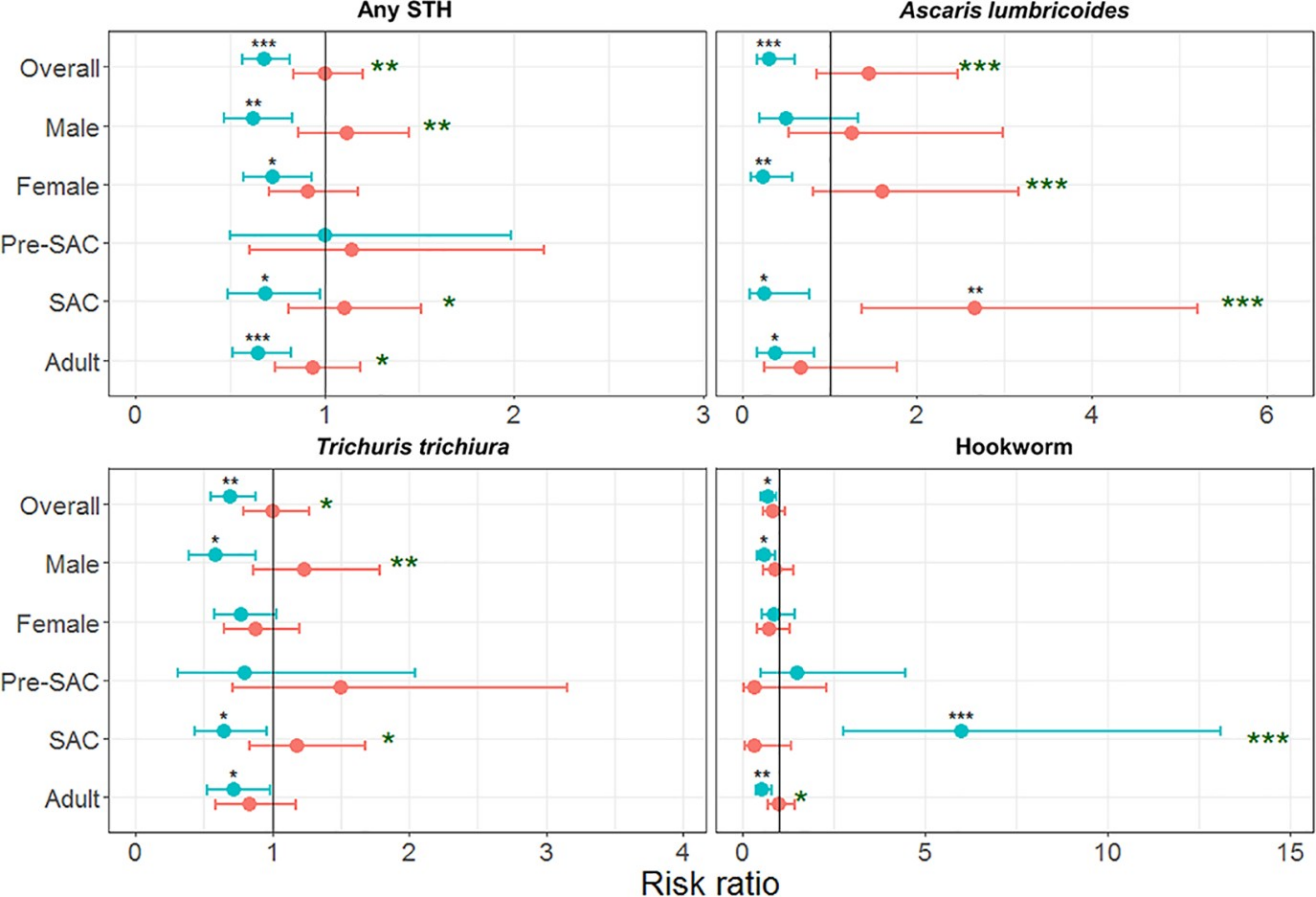
Risk ratios of STH prevalence between surveys. Blue = 4 months reinfection (survey 1 to survey 2). Red = 6 months reinfection (survey 2 to survey 3). * P ≤ 0.05, ** P ≤ 0.01, *** P ≤ 0.001 –black asterisks represent statistical significance of each risk ratio, green asterisks represent statistical difference between risk ratios for each group. Horizontal lines represent 95% confidence intervals. Pre-SAC = preschool-aged children (2–4 years old), SAC = school-aged children (5–14 years old), Adult = 15+ years old. No Ascaris lumbricoides infections were found in pre-SAC for all surveys, therefore no points are presented.

Risk ratios (RRs) for reinfection differed between STH species and reinfection period (Fig 2 and S1 Table). Four months post-MDA, the risk of infection was lower than in the preceding survey for all STHs (any STH RR = 0.67, 95% CI 0.56–0.81; *A*. *lumbricoides* RR = 0.31, 95% CI 0.16–0.59; *T*. *trichiura* RR = 0.70, 95% CI 0.55–0.88; hookworm RR = 0.69, 95% CI 0.50–0.95). The only statistically significant six-month RR was for *A*. *lumbricoides* infection in SAC (RR = 2.67, 95% CI 1.37–5.21). However, the six-month RRs were significantly higher than the four-month RRs for infection with all STH species (*P* < 0.05) except hookworm (*P* = 0.44). Six-month RRs were also statistically significantly higher than four-month RRs in SAC for infection with any STH, *A*. *lumbricoides* and *T*. *trichiura*, but were significantly lower for hookworm (*P* < 0.05). Six-month RRs were significantly higher in males for any STH and *T*. *trichiura* (*P* < 0.01) but higher in females for *A*. *lumbricoides* (*P* < 0.001).

From S1 to S2, 86.2% of *A*. *lumbricoides* infections, 52.8% of *T*. *trichiura* infections and 66.7% of hookworm infections were “cured” (Table 3). This decreased to 44.4% for *A*. *lumbricoides*, 50.0% for *T*. *trichiura* and 62.9% for hookworm infections from S2 to S3. Whereas, from S1 to S2, 1.0%, 4.6% and 3.8% of previously uninfected people became infected with *A*. *lumbricoides*, *T*. *trichiura* and hookworm infections, respectively. From S2 to S3, 1.6%, 6.7% and 3.3% of previously uninfected people became infected with *A*. *lumbricoides*, *T*. *trichiura* and hookworm infections, respectively.

**Table 3:** Percentage and number (n) of individual infections between infection and intensity groups (overall n = 523).

	*Ascaris lumbricoides*		*Trichuris trichiura*		Hookworm	
	S1-S2	S2-S3	S1-S2	S2-S3	S1-S2	S2-S3
**Never infected (neg-neg)**	93.50 (489)	96.75 (506)	79.16 (414)	82.22 (430)	86.81 (454)	90.25 (472)
**Cured (pos-neg)**	4.78 (25)	0.76 (4)	8.99 (47)	5.93 (31)	6.50 (34)	4.21 (22)
**Became infected (neg-pos)**	0.96 (5)	1.53 (8)	3.82 (20)	5.93 (31)	3.44 (18)	3.06 (16)
**Decrease intensity group (pos-pos)**	0.38 (2)	0	0.96 (5)	0.38 (2)	0.19 (1)	0
**Same intensity group (pos-pos)**	0.19 (1)	0.57 (3)	6.88 (36)	4.97 (26)	3.06 (16)	2.49 (13)
**Increase intensity group (pos-pos)**	0.19 (1)	0.38 (2)	0.19 (1)	0.57 (3)	0	0

Pos = positive. Neg = negative. S1 = survey 1 (August 2015), S2 = survey 2 (December 2015), S3 = survey 3 (June 2016).

### Reinfection–intensity

Mean EPG significantly decreased for *A*. *lumbricoides* (*P* < 0.01) and *T*. *trichiura* (*P* < 0.0001) from S1 to S2, and significantly increased for hookworm (*P* < 0.01). No changes in EPG from S2 to S3 were statistically significant. Over the year (S1 to S3), mean EPG significantly decreased for *T*. *trichiura* (*P* < 0.01) and hookworm (*P* < 0.001). The increase in *A*. *lumbricoides* EPG was not statistically significant (Table 2). A majority of the STH infections in all three surveys were low intensity infections for *T*. *trichiura* and hookworm (88.7–93.5% and 97.1–100%, respectively), whereas the majority of *A*. *lumbricoides* infections were low intensity in S2 (55.6%) and medium intensity in S1 (51.7%) and S3 (53.8%). At the final survey (S3) there were no medium/high intensity hookworm infection in SAC but 6.14% and 5.26% *A*. *lumbricoides* and *T*. *trichiura* infections, respectively. Therefore, the WHO target of less than 1% MHII in SAC has not been achieved in the study villages.

**Fig 3:**
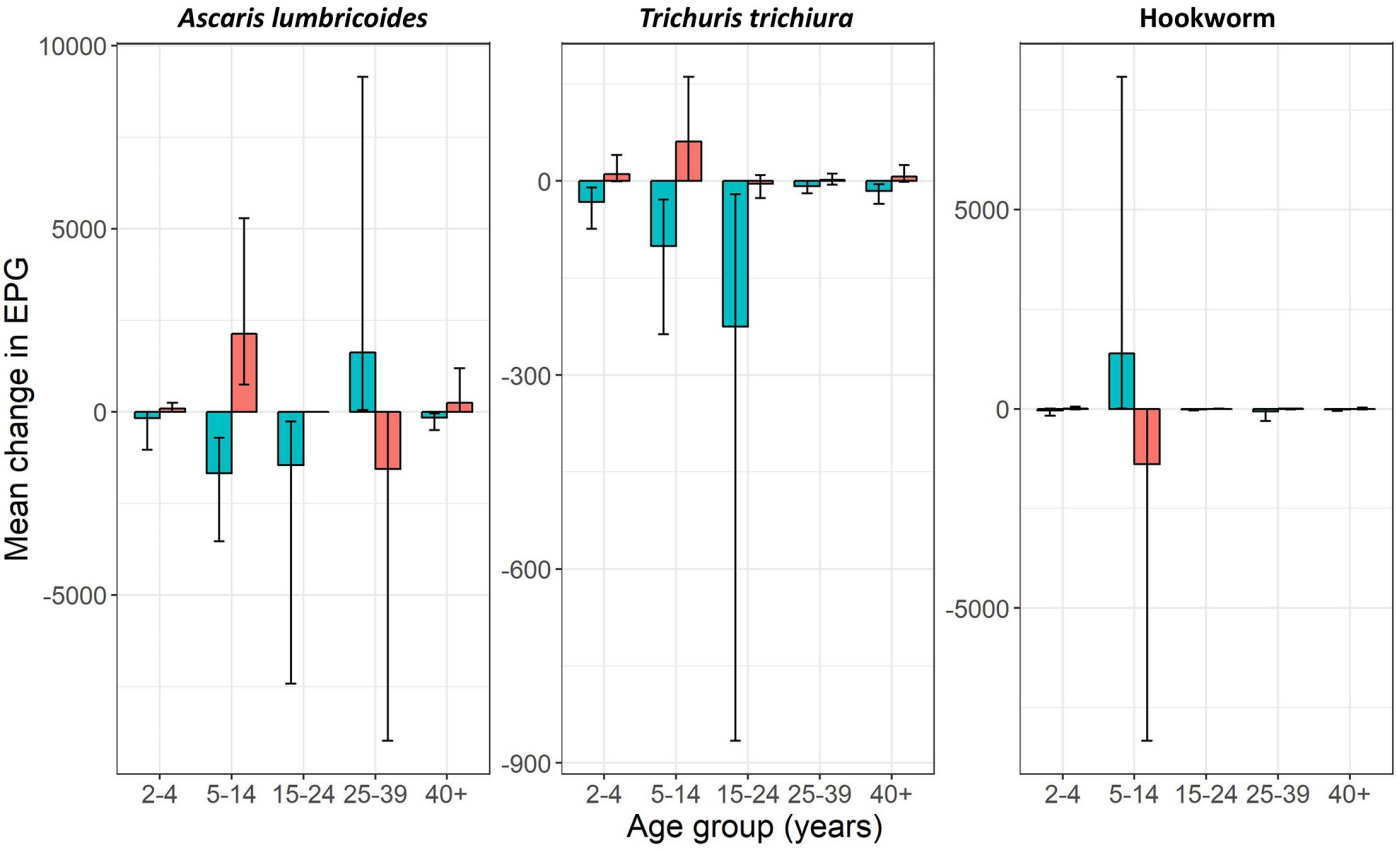
Mean change in eggs per gram of faeces (EPG) by age group. Blue bars = 4 months reinfection (survey 1 to survey 2). Red bars = 6 months reinfection (survey 2 to survey 3). Vertical lines represent 95% confidence intervals.

When mean EPG change was stratified by age group (Fig 3 and S2 Table) mean *A*. *lumbricoides* and *T*. *trichiura* EPG decreased from S1 to S2 and increased from S2 to S3 for all age groups except the 25–39 year olds for *A*. *lumbricoides* for which the opposite occurred. The mean change in EPG was not homogenous between all age groups. There was minimal change in mean EPG for both *A*. *lumbricoides* and *T*. *trichiura* in the youngest and oldest age groups. For hookworm, the increase and decrease in mean EPG was driven by the change in 5–14 year olds. A particularly high hookworm EPG result in the SAC group (158,136 EPG) for S2 may have skewed these results upwards.

### Predisposition to STH infection (consistent infection)

A total of 38 (7.27%) participants had STH infections in all three surveys and 67 (12.81%) had infections for any two of the three surveys. Correlation coefficients (Kendall Tau-b) of individual participants’ egg count results between surveys were statistically significant for all species of STH (Table 4). Most of the correlations remained significant when stratified by sex or age group. *Trichuris trichiura* egg counts had the strongest concordance between surveys especially for males, SAC and pre-SAC. The strongest concordance was for hookworm egg counts in pre-SAC between the first and second surveys. However, the Kendall’s Tau-b value may have been inflated due to the small number of pre-SAC infected with hookworm (two in survey one, three in survey two). Non-significant and low Tau values were calculated for *A*. *lumbricoides* infection in males and hookworm infection in SAC, denoting little evidence for predisposition in these groups.

**Table 4:** Kendall’s Tau-b correlation coefficients for individual participants’ egg counts between surveys.

		*Ascaris lumbricoides*			*Trichuris trichiura*			Hookworm		
		S1-S2	S2-S3	S1-S3	S1-S2	S2-S3	S1-S3	S1-S2	S2-S3	S1-S3
Overall		0.23***	0.45***	0.23***	0.50***	0.43***	0.48***	0.36***	0.36***	0.37***
Sex	Male	0.02	0.44***	0.14**	0.54***	0.50***	0.58***	0.41***	0.43***	0.42***
	Female	0.37***	0.45***	0.27***	0.47***	0.38***	0.40***	0.27***	0.27***	0.25***
Age group	Pre-SAC	NA	NA	0.03	0.62***	0.55***	0.41*	0.81***	0.57***	0.67***
	SAC	0.30*	0.58***	0.23*	0.62***	0.41***	0.56***	0.02	0.25*	0.01
	Adults	0.18***	0.40***	0.24***	0.40***	0.40***	0.38***	0.38***	0.37***	0.36***

* P ≤ 0.05. ** P ≤ 0.01. *** P ≤ 0.001. S1 = survey 1 (August 2015). S2 = survey 2 (December 2015). S3 = survey 3 (June 2016). Kendall Tau-b values: Blue = 0.00–0.25, Green = 0.26–0.50, Orange = 0.51–0.75, Red = 0.76–1.00, Grey = non-significant. Pre-SAC = preschool-aged children (2–4 years old). SAC = school-aged children (5–14 years old). Adults = 15+ years old. NA = comparison not possible (zero Ascaris lumbricoides infections in pre-SAC survey 2).

## Discussion

MDA programmes have been ongoing in the delta region of since 2003 for STH, and since 2013 for LF [10,12]. Whilst STH prevalence has dropped significantly since the initiation of MDA, the prevalence target set by WHO to discontinue MDA (under 1%) has not yet been reached in surveyed communities [14,28]. Currently, there is no monitoring and evaluation (M&E) of STH in Myanmar and no longitudinal studies have taken place since 1990 [35]. It is therefore important for longitudinal M&E studies to take place in the country so that the long-term impact of MDA can be evaluated. The results of this study show that overall STH prevalence was significantly reduced following two community-wide MDA rounds of the study sample (27.92 to 18.93%), and the intensities of infection (measured by EPG) of *T*. *trichiura* and hookworm were significantly reduced (73.56 to 41.07 EPG and 40.2 to 11.47 EPG, respectively). However, in the final survey, prevalence of STH was 28.07% in SAC, indicating that, according to the WHO guidelines, MDA should continue at the current frequency [4].

In this analysis, risk ratios were used to describe the patterns of infection over a four-month and six-month reinfection period. RRs for the six-month reinfection period were significantly higher than for the four-month reinfection period. This is not surprising since the extra two months allows more time for people to become newly infected and for surviving infections to re-establish. If we assume that the MDA rounds had cleared infection, then the data suggest that, in the study setting, four months is not enough time for STH to re-infect individuals to the prevalence levels pertaining before that particular round of treatment, but six months may be enough time. However, it is more likely that, due to sub-100% drug efficacy plus non-compliance to treatment, some infections were retained after MDA, and six months was enough time for the surviving helminths to release sufficient eggs to trigger the acquisition of new *A*. *lumbricoides* infections in SAC. Due to the low efficacy of albendazole against *T*. *trichiura* (as low as 30.7% and 43.6% cure rate in meta-analyses by Moser *et al*. [36] and Keiser *et al*. [17], respectively), it is possible that there was a greater magnitude of reestablishment of surviving worms for this species as proportionally fewer *T*. *trichiura* worms will have been affected by the initial treatment and thus more *T*. *trichiura* worms will have survived to reproduce. In the study sites included in this analysis, *T*. *trichiura* is the most prevalent STH in all age groups (peak of 29.82% in SAC, S1). Whilst shortening the time between MDA rounds may limit reestablishment of all STH species, if a single dose of albendazole is the treatment strategy in STH MDA programmes, the prevalence of *T*. *trichiura* will not be adequately reduced [37,38]. Research is ongoing into the efficacy of co-administrations with anthelminthics such as moxidectin, ivermectin or tribendimidine to target *T*. *trichiura* [39–41]. Helminth eggs are highly durable in the right environmental conditions. *A*. *lumbricoides* and *T*. *trichiura* eggs can remain viable and infective for several months [42], so individuals can become reinfected from eggs persisting in the environment, without the need for the deposition of new infective stages [16,43]. There is also the possibility of a seasonal effect on transmission [44,45]. The first and third surveys both took place during the dry season, whereas the second survey took place during the rainy season. Infective stage survival is known to be increased during rainy seasons [46–48]. It should also be noted that, for logistical reasons, the third survey in this study (June 2016) took place two months prior to the usual timing of the STH MDA round (August) (Fig 1). During the annual August MDA round, only children aged 2–9 years old are treated. As such, all 2–9 year olds not enrolled in the study will have had a further two months for reinfection and all other age groups not enrolled in the study will have a further six or seven months until the community-wide lymphatic filariasis (LF) MDA round (through GPELF). Therefore, we can hypothesise that if six months is enough time for STH prevalence to re-establish to the levels immediately prior to the last MDA round, the eight months between treatments for SAC and the 12 months between treatments for all other age groups may also allow infection to fully re-establish.

Prevalence is used as a key STH epidemiological metric, but intensity of infection is more important as a determinant of morbidity [2]. Whilst STH prevalence dropped significantly between the first and third surveys, the slight reductions in mean *T*. *trichiura* and hookworm EPG were not statistically significant. STH intensity at the beginning of the study was already at a very low level. Most participants infected with STH had low intensity infections. However, at S3 6.14% of SAC had MHII of *A*. *lumbricoides* and 5.26% with *T*. *trichiura*. The WHO target is to decrease MHII in SAC to below 1%, thereby indicating that STH in that country has been eliminated as a public health problem [2]. Unfortunately, this has not yet been achieved in the study villages. Prior work on the effect of long-term MDA programmes on STH have identified that substantial drops in STH prevalence and intensity in the first years of MDA may be followed by smaller reductions in subsequent years [49,50]. For example, an eight year MDA programme in Burundi reported significant drops in prevalence in the first four years and no further decrease in the last four years [50]. A monitoring survey in Kenya found that the largest reductions in *A*. *lumbricoides* and hookworm prevalence and intensity were recorded after the first MDA in comparison to the second MDA and whilst prevalence of *T*. *trichiura* was significantly reduced after three years of MDA, the proportion of medium to high *T*. *trichiura* infections was not [51]. The reasons for this may well be related to MDA coverage levels and individual compliance to treatment at multiple rounds of treatment [52]. Few studies to date have recorded individual compliance to treatment but persistent low prevalence may, in part, be due to persistent non-compliers to treatment [53]. The possibility of emerging resistance against anthelminthics should also continue to be considered and monitored, especially in mass treatment programmes that have been ongoing for an extended period of time [54,55].

Evidence of predisposition to STH infection has been found in several epidemiological studies [22,56,57] and the results of the Kendall’s Tau-b analysis indicates that predisposition to infection exists within the study sample. Concordance between egg counts indicates that the same individuals have reinfected with STH after treatment. Stronger concordance between survey egg counts, and therefore stronger evidence for predisposition, was found in males and the younger age groups for *T*. *trichiura* and hookworm infection but only in females for *A*. *lumbricoides* infection. This is in agreement with Holland *et al*. [58], who found stronger evidence for *A*. *lumbricoides* predisposition in females, but in disagreement with Haswell-Elkins *et al*. [59] and Quinnell *et al*. [60], with females more predisposed to hookworm infection. Whilst identifying individual people that are predisposed to infection would be costly and time-consuming, requiring diagnosis at several time points, building and collating evidence on groups of people (e.g. based on sex, age or occupation) that are predisposed to infection would allow them to be prioritised in MDA programmes when possibly targeting transmission interruption [61,62]. The reasons that underlie predisposition to infection (genetic, immunological, behavioural or exposure-related) are difficult to separate and quantify in epidemiological studies. The “susceptibility” factors of predisposition (genetics and immunology) are unlikely to change with public health interventions, but the “exposure” factors can be reduced through health education and WaSH initiatives.

A limitation of this study related to the data collection study is the low sensitivity of the Kato-Katz technique as a diagnostic tool. It is highly possible that infections were missed due to its use [63]. Another limitation is that, for important ethical reasons, the whole study sample (all ages) had to be treated during the MDA rounds that immediately followed the surveys, instead of the usual targeted ages (SAC only after the first and third surveys). The patterns of reinfection presented here therefore do not necessarily represent the patterns that will have occurred in previous years, during routine MDA. As we could not confirm clearance of infection after MDA, the results may not be viewed as true “reinfection”. However, the aim of individual treatment during MDA programmes is not to clear infection in hosts but to reduce the intensity of infection and subsequent morbidity [4,64]. Therefore, the “reinfection” we measure in this study is synonymous with the reinfection that is occurring between treatments in most endemic country MDA programmes. During data collection we attempted to ensure that treatment was taken via directly-observed therapy (DOT) where possible, but without data to confirm that infections were cleared we cannot assume this was always the case. Finally, this analysis focuses on the individuals that were present for all three surveys during the epidemiological study. It is possible that these individuals may have systematically different behaviour to those that dropped out or were lost to follow-up.

The key epidemiological observation in this study is the persistence of infection despite frequent and community wide MDA (through the STH MDA programme and GPELF), and the strong evidence for predisposition. Whilst the majority of infections were low intensity, the target of below 1% MHII in SAC has not been achieved and community-wide prevalence of STH at the end of the study was 18.93%. The prevalence of any STH in SAC at the end of the study (S3) was 28.07% which exceeds the threshold that WHO recommends for stopping MDA against STH [4]. Another key finding, from risk ratio analysis, is that a six-month gap between MDA rounds may be sufficient for STH infection to re-establish to pre-treatment levels within the community. In the long term, if diagnosis can be made more precise with new tools such as qPCR, and the costs of such tests greatly reduced, then future STH control may need to be based on targeted treatment to those predisposed to infection in order to eliminate transmission [65,66].

## Supporting information

S1 Fig Flow-chart of study participation. (TIF)

S1 Table Risk ratios of STH infection between surveys. (XLSX)

S2 Table Mean change in eggs per gram of faeces (EPG) between surveys. (XLSX)

S1 File STROBE checklist. (DOC)
