# Supplementary figures and images for: Soil-transmitted helminth reinfection four and six months after mass drug administration: results from the delta region of Myanmar
Source: PLoS Negl Trop Dis. 2019 Feb 15;13(2):e0006591. doi: 10.1371/journal.pntd.0006591 (PMC6395004; doi:10.1371/journal.pntd.0006591)

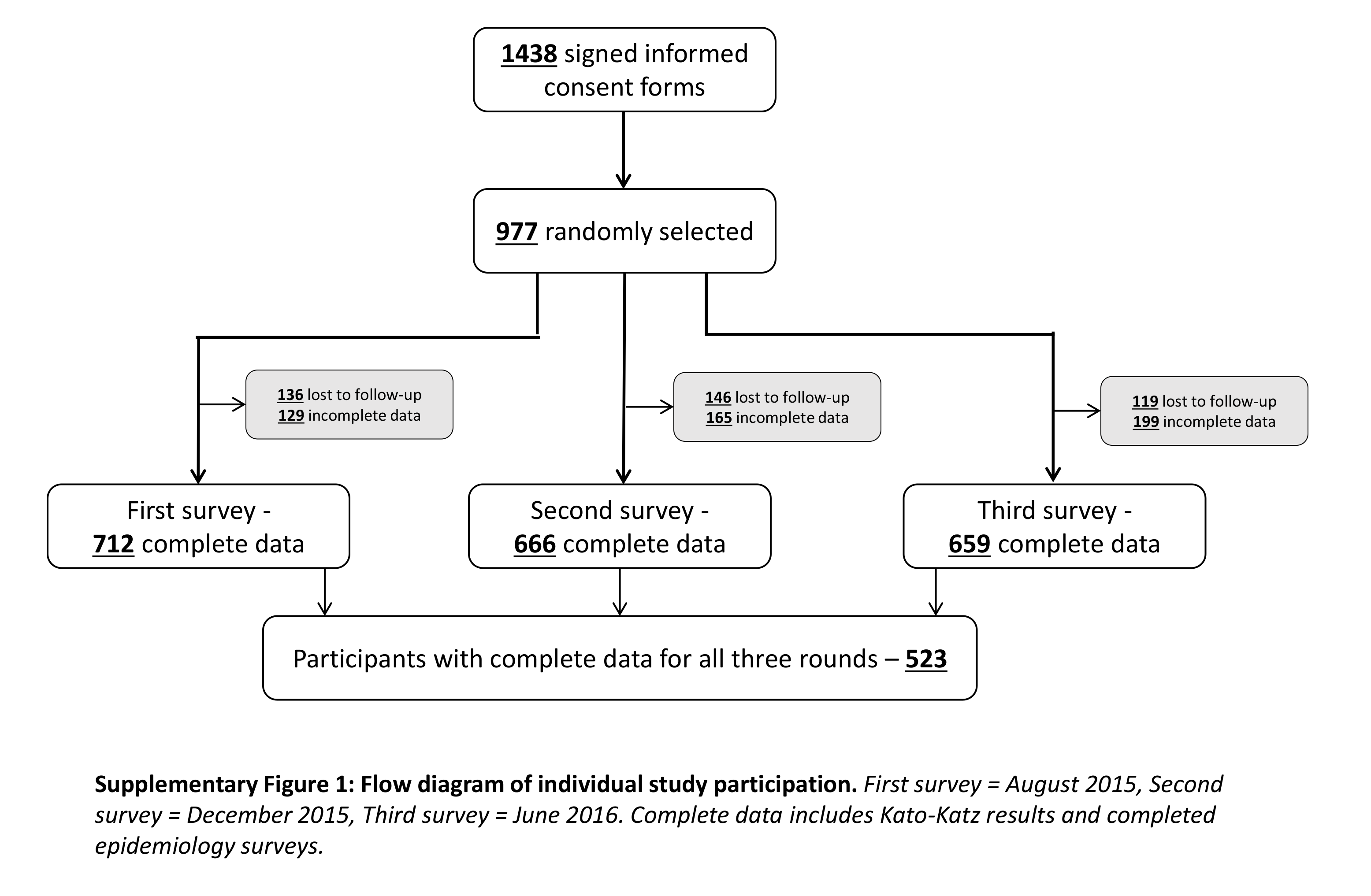

Supplement: S1 Fig — (TIF) [file pntd.0006591.s001.tif]
