# Supplementary material for: Soil-transmitted helminth reinfection four and six months after mass drug administration: results from the delta region of Myanmar
Source: PLoS Negl Trop Dis. 2019 Feb 15;13(2):e0006591. doi: 10.1371/journal.pntd.0006591 (PMC6395004; doi:10.1371/journal.pntd.0006591)
Supplement: S1 File — (DOC) [file pntd.0006591.s004.doc]

STROBE Statement—checklist of items that should be included in reports of observational studies

|  | Item No | Recommendation |
| --- | --- | --- |
| **Title and abstract** | 1 | (*a*) Indicate the study’s design with a commonly used term in the title or the abstract p2 –abstract (“epidemiological study”, “three surveys”) |
|  |  | (*b*) Provide in the abstract an informative and balanced summary of what was done and what was found p2 – “data from an epidemiological study on STH, comprising three surveys conducted between June 2015 and June 2016 in the delta region of Myanmar, are analysed to determine how STH prevalence and intensity in the study community changes over the course of a year, including reinfection after two MDA rounds in which the whole study sample (all age groups, n=523) were treated with albendazole” |
| Introduction | | |
| Background/rationale | 2 | Explain the scientific background and rationale for the investigation being reported p4-5 – Myanmar is endemic for STH infection and has been treating school-aged children with albendazole as part of a mass drug administration programme for approximately 10 years. There have been no longitudinal studies on STH in Myanmar since 1990. |
| Objectives | 3 | State specific objectives, including any prespecified hypotheses p5 – “The aim of this analysis is to determine how the prevalence and intensity of STH infection changes over the course of a year” |
| Methods | | |
| Study design | 4 | Present key elements of study design early in the paper p6-7, Dunn *et al.* reference, Fig 1 – “Data were collected in an STH epidemiological study that has been detailed in a previous publication...” |
| Setting | 5 | Describe the setting, locations, and relevant dates, including periods of recruitment, exposure, follow-up, and data collection p6-7 |
| Participants | 6 | (*a*) *Cohort study*—Give the eligibility criteria, and the sources and methods of selection of participants. Describe methods of follow-up  *Case-control study*—Give the eligibility criteria, and the sources and methods of case ascertainment and control selection. Give the rationale for the choice of cases and controls  *Cross-sectional study*—Give the eligibility criteria, and the sources and methods of selection of participants p6-7 |
|  |  | (*b*) *Cohort study*—For matched studies, give matching criteria and number of exposed and unexposed  *Case-control study*—For matched studies, give matching criteria and the number of controls per case |
| Variables | 7 | Clearly define all outcomes, exposures, predictors, potential confounders, and effect modifiers. Give diagnostic criteria, if applicable p7-8 |
| Data sources/ measurement | 8* | For each variable of interest, give sources of data and details of methods of assessment (measurement). Describe comparability of assessment methods if there is more than one group p6-7 |
| Bias | 9 | Describe any efforts to address potential sources of bias Pg 7 – “random selection of households”. pg 19 – “we attempted to ensure that treatment was taken via directly-observed therapy where possible” |
| Study size | 10 | Explain how the study size was arrived at p7 |
| Quantitative variables | 11 | Explain how quantitative variables were handled in the analyses. If applicable, describe which groupings were chosen and why p8-9 |
| Statistical methods | 12 | (*a*) Describe all statistical methods, including those used to control for confounding p8-9 – Statistical analysis section |
|  |  | (*b*) Describe any methods used to examine subgroups and interactions |
|  |  | (*c*) Explain how missing data were addressed |
|  |  | (*d*) *Cohort study*—If applicable, explain how loss to follow-up was addressed  *Case-control study*—If applicable, explain how matching of cases and controls was addressed  *Cross-sectional study*—If applicable, describe analytical methods taking account of sampling strategy |
|  |  | (*e*) Describe any sensitivity analyses |

Continued on next page

| Results | | |
| --- | --- | --- |
| Participants | 13* | (a) Report numbers of individuals at each stage of study—eg numbers potentially eligible, examined for eligibility, confirmed eligible, included in the study, completing follow-up, and analysed p7 – “overall 523 participants from 211 households...” |
|  |  | (b) Give reasons for non-participation at each stage |
|  |  | (c) Consider use of a flow diagram S1 Figure |
| Descriptive data | 14* | (a) Give characteristics of study participants (eg demographic, clinical, social) and information on exposures and potential confounders Table 1 |
|  |  | (b) Indicate number of participants with missing data for each variable of interest |
|  |  | (c) *Cohort study*—Summarise follow-up time (eg, average and total amount) Fig 1. P7 – “the study comprised three parasitology surveys in August 2015, December 2015 and June 2016” |
| Outcome data | 15* | *Cohort study*—Report numbers of outcome events or summary measures over time. Table 1 |
|  |  | *Case-control study—*Report numbers in each exposure category, or summary measures of exposure |
|  |  | *Cross-sectional study—*Report numbers of outcome events or summary measures |
| Main results | 16 | (*a*) Give unadjusted estimates and, if applicable, confounder-adjusted estimates and their precision (eg, 95% confidence interval). Make clear which confounders were adjusted for and why they were included Table 2, Fig 2, Fig 3, S2 Table, S3 Table |
|  |  | (*b*) Report category boundaries when continuous variables were categorized p8-9 |
|  |  | (*c*) If relevant, consider translating estimates of relative risk into absolute risk for a meaningful time period |
| Other analyses | 17 | Report other analyses done—eg analyses of subgroups and interactions, and sensitivity analyses |
| Discussion | | |
| Key results | 18 | Summarise key results with reference to study objectives p15 – “The results of this study show that overall STH prevalence was significantly reduced following two community-wide MDA rounds of the study sample…” |
| Limitations | 19 | Discuss limitations of the study, taking into account sources of potential bias or imprecision. Discuss both direction and magnitude of any potential bias p18-19 – “A limitation of this study...is the low sensitivity of the Kato-Katz technique...could not confirm clearance of infection...” |
| Interpretation | 20 | Give a cautious overall interpretation of results considering objectives, limitations, multiplicity of analyses, results from similar studies, and other relevant evidence p19 |
| Generalisability | 21 | Discuss the generalisability (external validity) of the study results p19 – “future STH control may need to be based on targeted treatment to those predisposed to infection...” |
| Other information | | |
| Funding | 22 | Give the source of funding and the role of the funders for the present study and, if applicable, for the original study on which the present article is based Funding information |

*Give information separately for cases and controls in case-control studies and, if applicable, for exposed and unexposed groups in cohort and cross-sectional studies.

**Note:** An Explanation and Elaboration article discusses each checklist item and gives methodological background and published examples of transparent reporting. The STROBE checklist is best used in conjunction with this article (freely available on the Web sites of PLoS Medicine at http://www.plosmedicine.org/, Annals of Internal Medicine at http://www.annals.org/, and Epidemiology at http://www.epidem.com/). Information on the STROBE Initiative is available at www.strobe-statement.org.
